# Peanut Aflatoxin: Impact of Postharvest Insect Infestation and Storage Systems

**DOI:** 10.3390/insects15110836

**Published:** 2024-10-25

**Authors:** George N. Mbata, James K. Danso, Raegan L. Holton

**Affiliations:** 1 Entomology Research Laboratory, Agricultural Research Station, Fort Valley State University, 1005 State University Drive, Fort Valley, GA 31030, USA; james.danso@fvsu.edu; 2 Premium Peanut, 311 Barrington Road, Douglas, GA 31535, USA; raegan.holton@premiumpnut.com

**Keywords:** postharvest system, insect pests, aflatoxin, food safety, farmers stock peanut

## Abstract

Effective postharvest management is crucial for the U.S. peanut industry, given the humid-subtropical climate that promotes insect pests, *Aspergillus* mold, and aflatoxin contamination in peanut-producing states. This study surveyed five conventional metal warehouses (CMWs) and thirteen flat storage facilities (FSFs) in northeastern, southeastern, and southwestern Georgia, USA, from December 2022 to July 2023. Peanuts were sampled monthly to assess the temporal abundance and diversity of storage insect pests, damage variables, and aflatoxin levels in farmers’ stock peanuts. A monoclonal antibody-based affinity chromatographic assay determined the total aflatoxin levels in samples. The most common insect species, accounting for ~85.73 of the total captured, included *Cryptolestes ferrugineus*, *Tribolium castaneum*, *Ahasverus advena*, *Plodia interpunctella*, *Cadra cautella*, *Ephestia elutella, Liposcelis* spp., and *Lachesilla pedicularia*. Insect populations peaked in June and July, with similar diversity trends across CMWs and FSFs. Insect-damaged kernels, weight loss, discolored kernels, loose-shelled kernels, moisture content, and aflatoxin levels ranged from 3.02 to 11.45%, 1.11 to 3.60%, 2.11 to 11.20%, 1.56 to 4.91%, 6.89 to 8.85%, and 0.43 to 27.59 ppb, respectively. The high prevalence of insect pests, their damage capabilities, and the aflatoxin levels of infested peanuts highlight the need for an improved IPM strategy in warehouses to ensure safe and sustainable peanut storage in the USA.

## Simple Summary

This study investigates how storage systems, insect pest infestations, and environmental conditions impact aflatoxin contamination and peanut quality in the southeastern United States. It aims to identify key factors affecting the deterioration of peanut quality and aflatoxin levels in commonly utilized storage facilities for in-shell peanuts. The results indicated that peanuts stored in conventional metal warehouses had higher temperatures and moisture content, leading to increased insect infestations, and aflatoxin contamination compared to those stored in flat storage facilities. A significant correlation was found between elevated temperatures, equilibrium moisture content, and the proliferation of insect pests, which contributed to considerable quality degradation. The findings underscore the critical importance of maintaining optimal storage conditions in storage facilities to preserve peanut quality and prevent aflatoxin contamination, with implications for reducing economic losses and safeguarding public health from aflatoxin exposure.

## 1. Introduction

Globally, the quality, safety, and sustained availability of stored durable agricultural products are significantly influenced by postharvest storage conditions [1,2]. Peanuts (*Arachis hypogaea* L.), a key agricultural commodity, are susceptible to a diverse array of biotic and abiotic factors during storage, resulting in significant postharvest losses. Insect pest infestations and microbial contamination are mostly implicated in peanut postharvest losses. In the United States, the Indianmeal moth, *Plodia interpunctella* (Hübner) (Lepidoptera: Pyralidae), the almond moth, *Cadra cautella* (Walker) (Lepidoptera: Pyralidae), and the red flour beetle, *Tribolium castaneum* (Herbst) (Coleoptera: Tenebrionidae), are the most economically important postharvest insect pests of peanuts [3,4]. Other insect pests that infest peanuts and pose substantial economic threat to postharvest management of peanuts in the United States include the cigarette beetle, *Lasioderma serricorne* (F.) (Coleoptera: Ptinidae), the corn sap beetle, *Carpophilus dimidiatus* (F.), the dried fruit beetle, *Carpophilus hemipterus* (L.) (Coleoptera: Nitidulidae), the tobacco moth, *Ephestia elutella* (Hübner), the Mediterranean flour moth, *Ephestia kuehniella* (Zeller) (Lepidoptera: Pyralidae), the merchant grain beetle, *Oryzaephilus mercator* (Fauvel), the sawtoothed grain beetle, *Oryzaephilus surinamensis* (L.), the foreign grain beetle, *Ahasverus advena* (Waltl) (Coleoptera: Silvanidae), the confused flour beetle, *Tribolium confusum* Jacquelin du Val, the depressed flour beetle, *Palorus subdepressus* (Wollaston) (Coleoptera: Tenebrionidae), the flat grain beetle, *Cryptolestes pusillus* (Schonherr) (Coleoptera: Laemophloeidae), the hairy fungus beetles, *Typhaea stercorea* (L.) (Coleoptera: Mycetophagidae), and the warehouse beetle, *Trogoderma variabile* Ballion (Coleoptera: Dermestidae) [5,6].

Losses in postharvest peanuts resulting from insect infestations can include both physical damage and quality deterioration due to contamination [4,7]. The presence of insects along with associated exuviae, feces, and cadavers, in shelled peanuts or peanut products, contributes to qualitative and quantitative losses [3,8]. Again, a significant infestation by insects has been demonstrated to strongly correlate with elevated temperatures and relative humidity levels within the storage systems [9]. These environmental conditions can induce discoloration, decay, oxidative rancidity, mold growth, and contamination of postharvest peanuts with aflatoxins [10]. Moreover, stored-product insect pests, particularly beetles and moths, serve as vectors for aflatoxigenic fungi, thereby facilitating aflatoxin contamination [11]. Thus, the constant migration of insect pests within storage systems serves as an effective mechanism for dispersing viable spores of mycotoxigenic fungi.

Aflatoxins (AFB_1_, AFB_2_, AFG_1_, and AFG_2_), a group of naturally occurring secondary metabolites, are primarily produced by various fungal species within the genus *Aspergillus*, notably *A. flavus* Link, *A. parasiticus* Speare, and *A. nomius* Kurtzman et al. [12,13]. Similar to infestations by stored-product insects, *Aspergillus* infections and subsequent aflatoxin production in peanuts can occur prior to postharvest operations and may exacerbate during storage, particularly under warm-humid conditions [9,14]. The presence of aflatoxin-contaminated peanuts within the supply chain poses significant health risks due to the potent carcinogenic properties of aflatoxins and their potential to induce acute or chronic health effects in both humans and animals [15]. Furthermore, aflatoxin contamination presents a dual threat, impacting not only public health but also trade dynamics, thereby posing a significant challenge to the economic sustainability of the U.S. peanut industry, especially when maximum total aflatoxin levels exceed regulatory limits. For example, in 2019, 30% of all shelled lots of U.S. peanuts were rejected at shelling plants due to high levels of aflatoxin residues, resulting in a significant financial loss of USD 126 million to the industry. Even in 2021, considered a relatively clean year, aflatoxin still imposed a financial burden of USD 58 million on the U.S. peanut industry [16]. Consequently, there is an urgent need to implement sustainable aflatoxin mitigation measures, particularly in postharvest peanuts, to safeguard consumer health and enhance the competitiveness of the U.S. peanut industry in both domestic and international markets.

There are several types of structures used for bulk-stored peanuts worldwide. In the United States, farmer stock warehouses, primarily conventional metal warehouses (CMWs) and flat storage facilities (FSFs), are commonly utilized for the commercial bulk storage of peanuts. These facilities present distinct storage conditions that can impact insect population dynamics, *Aspergillus* growth, and aflatoxin formation [17]. Conventional metal warehouses can create an environment conducive to the proliferation of storage pests due to their structural composition, mainly steel. Factors such as temperature, relative humidity, and dew point within these structures influence the population dynamics of insects, including beetles, moths, and psocids. Though differing in design, flat storage facilities (FSFs) can also sustain insect infestations, mold growth, and aflatoxin accumulation in stored commodities. Even with in-built mechanical or automated aeration control/ventilation systems, both types of storage facilities have distinct thermal and humidity profiles that can potentially affect insect pest activity, kernel rehydration, *Aspergillus* growth, and subsequent aflatoxin contamination [18]. Hence, understanding the interactions between biotic and abiotic factors within storage structures across various storage durations is imperative for devising effective aflatoxin mitigation strategies. Moreover, recognizing the challenges posed by different storage structures facilitates the development of targeted mitigation strategies, thereby ensuring the safety and quality of peanuts during postharvest storage. Primarily, obtaining comprehensive data on insect and mold-related variables, alongside thermo-hygrometric conditions that impact postharvest peanut quality within storage facilities, constitutes a fundamental prerequisite for the development of accurate risk assessment models and the formulation of targeted aflatoxin management strategies. Therefore, this study was aimed at assessing the temporal distribution and abundance of storage insect pests, related damage variables, and associated aflatoxin residues in farmer stock peanuts (FSP) stored in commercial warehouses (CMWs and FSFs) in Georgia State, USA. Additionally, the correlations between insect species, damaged variables, storage facility types, storage duration, environmental conditions, and aflatoxin residues in the farmer peanut stocks were established.

## 2. Materials and Methods

### 2.1. Experimental Location and Experimental Design

The experiment was conducted in farmer stock peanut (FSP) warehouses located at the buying points in the northeastern, southeastern, and southwestern areas of Georgia (GA), a major peanut production hub in the United States. Two types of storage structures were investigated: 5 conventional metal warehouses (CMWs) and 13 flat storage facilities (FSFs). Warehouses were filled with in-shell FSPs with capacities ranging from 3700 to 13,500 tons and 7938 to 19,845 tons for CMWs and FSFs, respectively. The loading of warehouses with in-shell peanuts typically occurs from late September to October of the production season. However, monitoring and sampling of in-shell peanuts stored in warehouses started in December 2022 through July 2023, corresponding to the initial sampling month (2–3 months after receiving in-shell peanuts) and bailing-out periods of warehouses (9–10 months following receipt of in-shell peanuts), respectively. Typically, storage facilities had runner peanut types (others: Spanish, Valencia, and Virginia types). Storage management and practices across all study locations were similar, with variations in storage structures, storage capacities, storage conditions, and fumigation regimes. Typically, phosphine fumigation was conducted 2–4 times in each warehouse during the storage period as needed to manage insect pest pressure and maintain the quality and safety of stored peanuts. Solid formulations of phosphine (pellets/tablets) were applied in sealed structures at a rate of 12–27 cases per working capacity (thus, the volume of the storage space being fumigated per cubic feet) over the 7-day exposure period. In both types of storage facilities, the first phosphine fumigation was conducted in January 2023 and the final in June 2023, a month prior to the removal of peanuts from the warehouses. The experimental data were analyzed statistically as a two-factor design. Factors were storage facility type (factor level 2: CMW and FSF) and sampling month (factor level 7: December 2022 and February, March, April, May, June, and July 2023). December to February (winter months), March to May (spring months), and June to August (summer months) represent the early-storage period, mid-storage period, and late-storage period, respectively, in Georgia, USA.

### 2.2. Warehouse Description

A conventional metal warehouse (CMW) in the USA for bulk storage of peanuts typically spans 100–300 feet (30.48–91.44 m) in length, 60–150 feet (18.29–45.72 m) in width, and stands 20–40 feet (6.10–12.19 m) tall. Constructed with corrugated metal (steel) walls and roofs, it ensures durability and weather resistance. The roof pitch is usually 12:12 (45°), allowing enough room in the headspace to install overhead belt conveyors and associated equipment to load peanuts into the warehouse [18]. The flooring is made of concrete for stability and easy cleaning. Ventilation is facilitated by strategically placed vents or fans along the walls or roof to remove excess heat and moisture from the peanuts by pulling outside air into the warehouse through the headspace while exhausting the moisture and heat-laden air above the peanuts and below the roof to the outside. In addition, the insulation and temperature control systems maintain optimal storage conditions. In a properly filled warehouse, peanuts will be 50–55 feet (15.24–16.76 m) deep under the ridge of the building and 23 feet (7.01 m) deep at the sidewall. In contrast, a flat storage facility (FSF) for bulk storage of peanuts in Georgia, USA, typically spans extensive lengths of 200–400 feet (60.96–121.92 m) and widths ranging from 100 to 200 feet (30.48 to 60.96 m), with heights typically 15 to 25 feet (4.57–7.62 m). Constructed with reinforced steel, FSFs offer durability and protection against environmental elements. The floor consists of durable concrete for stability and easy maintenance. The sampled FSFs are equipped with specialized ventilation systems designed to remove excess moisture from the headspace, maintaining optimal humidity levels and preventing mold growth. Additionally, temperature control mechanisms, such as insulation and cooling systems, may be incorporated to preserve peanut quality. Loading and unloading areas are equipped with wide doors at both ends to facilitate efficient access for trucks to load and unload the warehouses. Peanuts were transferred from trucks to stores using telescoping conveyor belts [18]. Peanut depth is usually uniform throughout the storage period with few peaks and valleys and no deeper than approximately 13 feet (3.96 m). Generally, FSFs have few or no peanuts in contact with the steel walls of the structure which are covered with woody boards. The ambient temperature (°C) and relative humidity (r.h. %) in the warehouses were monitored hourly throughout the sampling periods using thermo-hygrometric sensors (HOBO data loggers: Onset Computers, Bourne, MA, USA). Other IPM techniques employed in the sampled warehouses include sanitation measures (removal of peanut spillage around warehouses before loading and after peanut removal), peanut grading and segregation, routine inspection and monitoring of infestations using non-baited sticky and probe traps, replacing interior wooden insulation boards in FSFs prior to loading, and screening ventilator and aeration outlets.

### 2.3. Sampling for Insects, Damage Assessment, and Determination of Total Aflatoxin Residues

Monitoring and assessing insect pest infestation, damage variables, and aflatoxin contamination were performed through monthly direct visual assessments of piled, in-shell peanuts within warehouses. Thus, representative samples were collected monthly from each warehouse, drawing from 25 to 50 distinct points within the peanut pile at varying depths (0.15–1.5 m). Each warehouse was divided into six and at least four subsamples were collected from each sextant. This was performed using both grain probes (72″ (1.828 m) Long Brass Open Handle Probe, Seedburo Equipment, Chicago, IL, USA) and hand scoops. These samples were then placed into Ziploc storage bags, each clearly labeled and measuring 39 cm × 25 cm. Specifically, six bags were used, each holding 1 kg of in-shell peanuts. Thus, a total of 6 kg of peanut pods was sampled from each warehouse per month. These samples were subsequently transported to the Entomology Research Laboratory at the Agricultural Research Station of Fort Valley State University (FVSU) in Fort Valley, GA, for processing. Each 1 kg peanut sample was carefully divided into 500 g subsamples, one subsample for insect identification, species abundance, and soundness parameters, and the other for purity assessment, which included total aflatoxin residue determination. Samples for purity assessment were stored in a refrigerator at <4 °C before the aflatoxin analysis.

#### 2.3.1. Assessment of Insect Pests and Soundness Parameters of Peanuts

Each 500 g subsample (6 replicates per warehouse) was sieved in weeks 1 and 4 following sampling to recover all live insects (adults) using the U.S.A. standard sieve series: sieve #10 (2 mm openings) and sieve #25 (0.71 mm openings) (Dual Manufacturing Co., Franklin Park, IL, USA). Insect species were identified using the Grain Research and Development Corporation Stored Grain Pests Identification Guide [19], and insect numbers were recorded. Subsequently, the sifted peanuts were combined and 3 replicates of 500 g subsamples per warehouse were assessed for losses resulting from insect feeding activities and mold growth that included percentage of insect-damaged pods (%IDP), cracked pods (%CP), loose-shell kernels (%LSK), pod weight loss (%PWL), insect-damaged kernels (%IDK), kernel weight loss (%KWL), and discolored kernels (%DK). Thus, each 500 g sample was poured onto a tray, and all pods were examined using a hand lens (10× magnification) to identify pods with holes created by insects or pods silked together by moths. These damaged pods were separated from undamaged pods, and the numbers of pods in each category were recorded for the estimation of %IDP and %PWL. Similarly, all pods showing physiological or mechanical cracks in the hulls were separated and used for the estimation of %CP. Also, the number of kernels free from the hull (LSK) per 500 g of peanut pods was recorded. Subsequently, all pods per 500 g of peanuts were shelled, and all kernels were examined using the hand lens to identify kernels showing insect feeding activities and those with visible *Aspergillus* growth, and these were recorded and used for the estimation of %IDK, %KWL, and %DK. Additionally, %LSK was estimated using the number of loose kernels per 500 g pods and the total number of kernels per 500 g pods sample. Furthermore, equilibrium kernel moisture content (%EMC) was estimated using the AMTAST Grain Moisture Meter (AMT 65C, AMTAST USA, Inc., Lakeland, FL, USA). Peanut soundness parameters were estimated using the formulas (a, b, c, d, and e) provided, and each variable was replicated three times per sampling month per warehouse.

##### Formulae for Estimation of Soundness Parameters of Peanuts

(a) Percent insect-damaged pod/kernel (%) = $\frac{N_{d}}{{(N}_{u}+{\;N}_{d})}\times 100$; {Where *N_d_* is the number of insect-damaged pod/kernel and *N_u_* is the number of undamaged pod/kernel per 500 g of peanuts}
(b) Percent weight loss (%) = $\frac{\left(W_{u}\times N_{d}\right)-(W_{d}\times N_{u})}{W_{u}\;(N_{d}+N_{u})}\times 100$; {Where *W_u_* is the weight of undamaged pod/kernel, *N_u_* is the number of undamaged pod/kernel, *W_d_* is the weight of insect-damaged pod/kernel, and *N_d_* is the number of insect-damaged pod/kernel per 500 g of peanut, Boxall [20]}
(c) Percent cracked pod (%) = $\frac{N_{c}}{T_{p}}\times 100$; {Where *N_c_* is the number of pods with physiological or mechanical cracked hull and *T_p_* is the total number of pods per 500 g of peanuts}
(d) Percent discolored kernel (%) = $\frac{N_{dk}}{T_{k}}\times 100$; {Where *N_dk_* is the number of discolored kernels due to *Aspergillus flavus* and related molds or insect damage and *T_k_* is the total number of kernels per 500 g of peanuts}
(e) Percent loose-shelled kernels (%) = $\frac{N_{lsk}}{T_{k}}\times 100$; {Where *N_lsk_* is the number of kernels free from the hull per 500 g of pods and *T_k_* is the total number of kernels per 500 g of peanut pods}.

#### 2.3.2. Determination of Total Aflatoxin Residues (ppb) in Peanuts

The detection and estimation of total aflatoxin residues (B_1_, B_2_, G_1_, and G_2_) in peanut samples was performed using the VICAM AflaTestTM mycotoxin testing procedures (VICAMTM, 34 Maple Street, Milford, MA, USA). AflaTest^®^ utilizes a monoclonal antibody-based immunoaffinity chromatographic assay, providing quantitative analysis of aflatoxins in test samples. The procedure involves four analytical steps: sample extraction, dilution and filtration, immunoaffinity column chromatography, and quantification of aflatoxin levels via fluorometric detection. A comprehensive description of this procedure has been previously published [21]. Additionally, complete instructions, methodologies, procedures, and reagents pertinent to raw-shelled peanuts are outlined in the manufacturer’s instruction manual (AflaTest Fluorometer Instruction Manual, pg. 38, Milford, MA, USA). Three samples per peanut warehouse were subjected to analysis per sampling month.

### 2.4. Statistical Analysis

Analysis of variance (ANOVA) was conducted using the Generalized Linear Mixed Model Procedure (Proc GLIMMIX) (SAS/STAT Software Version 9.4) to assess the effects of storage facility type and storage duration on the various response variables (insect species, insect-damaged pod, cracked pod, pod weight loss, loose-shelled kernel, insect-damaged kernel, kernel weight loss, discolored kernel, kernel moisture content, temperature, relative humidity, and aflatoxin residues) investigated. Proc GLIMMIX modeled the fixed effects of storage facility type, storage duration, and their interactions for each of the response variables with specified response distribution (∼Gaussian) in SAS/STAT. Where appropriate, square root or arcsine square root transformations were applied to count or percentage data, respectively, to correct for heterogeneity of variances and lack of normality in the response variables. Least square means were compared for significant effects using Tukey’s Honest Significant Difference test. All tests were performed at the nominal 0.05 level of significance. Means and standard errors for each response variable are reported in tables and figures and were estimated using the Means Procedure (Proc Means) in SAS/STAT (SAS/STAT Software Version 9.4). Again, Pearson correlation coefficients were estimated using the Correlation Procedure (Proc CORR) in SAS/STAT (SAS/STAT Software Version 9.4) at a nominal level of 0.05 to determine the strength of association between peanut aflatoxin residues and other response variables (insect species, soundness parameters, and ambient environmental storage conditions). Moreover, the distribution of aflatoxin in the two storage facility types was compared using the non-parametric t-test Procedure (Proc NPAR1WAY) in SAS/STAT (SAS/STAT Software Version 9.4).

## 3. Results

### 3.1. Peanut Moisture Content (%), Temperature (°C), and Relative Humidity (%) in Storage Facilities

**Figure 1:**
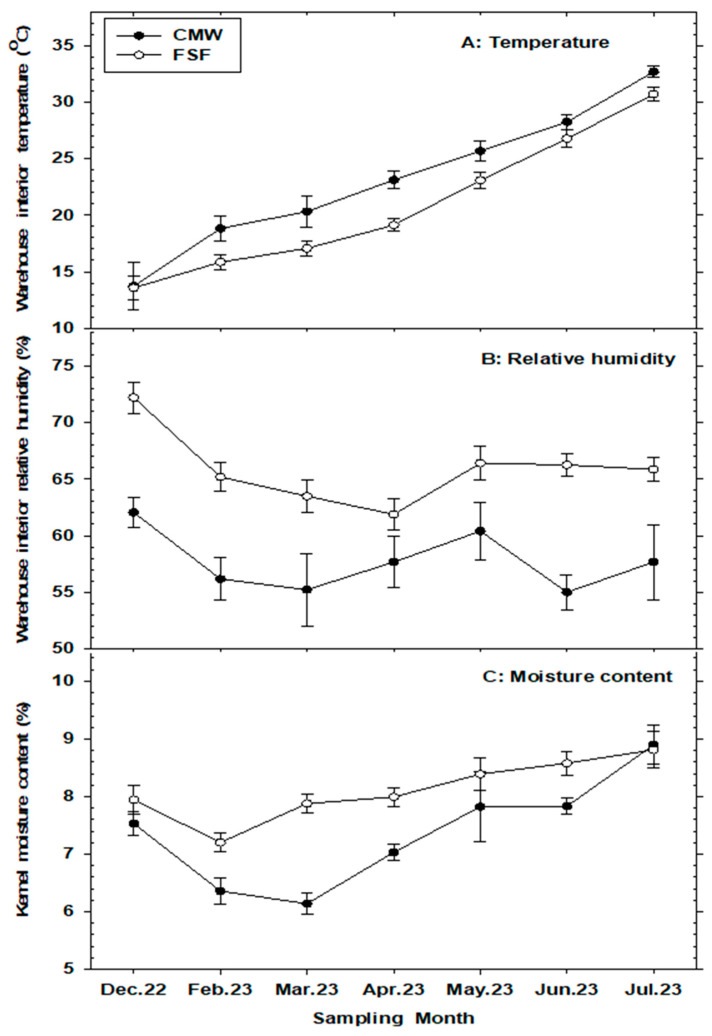
Mean (±SE) ambient temperature (°C) and relative humidity (%) monitored with HOBO data loggers in warehouses, and equilibrium moisture content (EMC, wet weight basis %) of peanuts stored in conventional metal warehouses (CMWs) and flat storage facilities (FSFs) in Georgia, USA during 2022–2023 storage period.

The main effects of the sampling month (hereafter referred to as ‘month’) and storage structure were significantly different (*p* < 0.05) for the environmental variables (ambient temperature and relative humidity) and the kernel moisture content of peanuts. However, their interactions were not significant (Table 1). Ambient temperature in warehouses was significantly and consistently higher in conventional metal warehouses (CMWs) compared to flat storage facilities (FSFs) throughout the monitoring months, except in December (the initial sampling month) (Figure 1A). There was a steady increase in temperature in both types of storage structures from the start of monitoring in December 2022 to July 2023, with monthly averages ranging from 13.73 to 32.67 °C and 13.57 to 30.69 °C in CMWs and FSFs, respectively. Conversely, ambient relative humidity (r.h.) in both storage facility types varied significantly across months and was considerably higher in the FSFs than in the CMWs, with monthly means ranging from 65.85 to 72.21% and 54.99 to 62.05%, respectively (Table 1 and Figure 1B). Similarly, the equilibrium moisture content (EMC, wet weight basis %) of peanuts fluctuated across months and was consistently higher in the FSFs compared to the CMWs, ranging from 7.20% to 8.81% and 6.14% to 7.83%, respectively (Table 1 and Figure 1C). Additionally, in both types of storage structures, the lowest and highest EMC were recorded in February 2023 and July 2023, respectively, corresponding to the winter and summer storage seasons. A statistically significant positive correlation was observed between the ambient temperature in storage structures and the EMC of peanuts (r = 0.85, *p* = 0.013), but not ambient r.h. (r = −0.19, *p* = 0.67).

### 3.2. Stored-Product Insect Pests in Farmer Stock Peanuts

**Figure 2:**
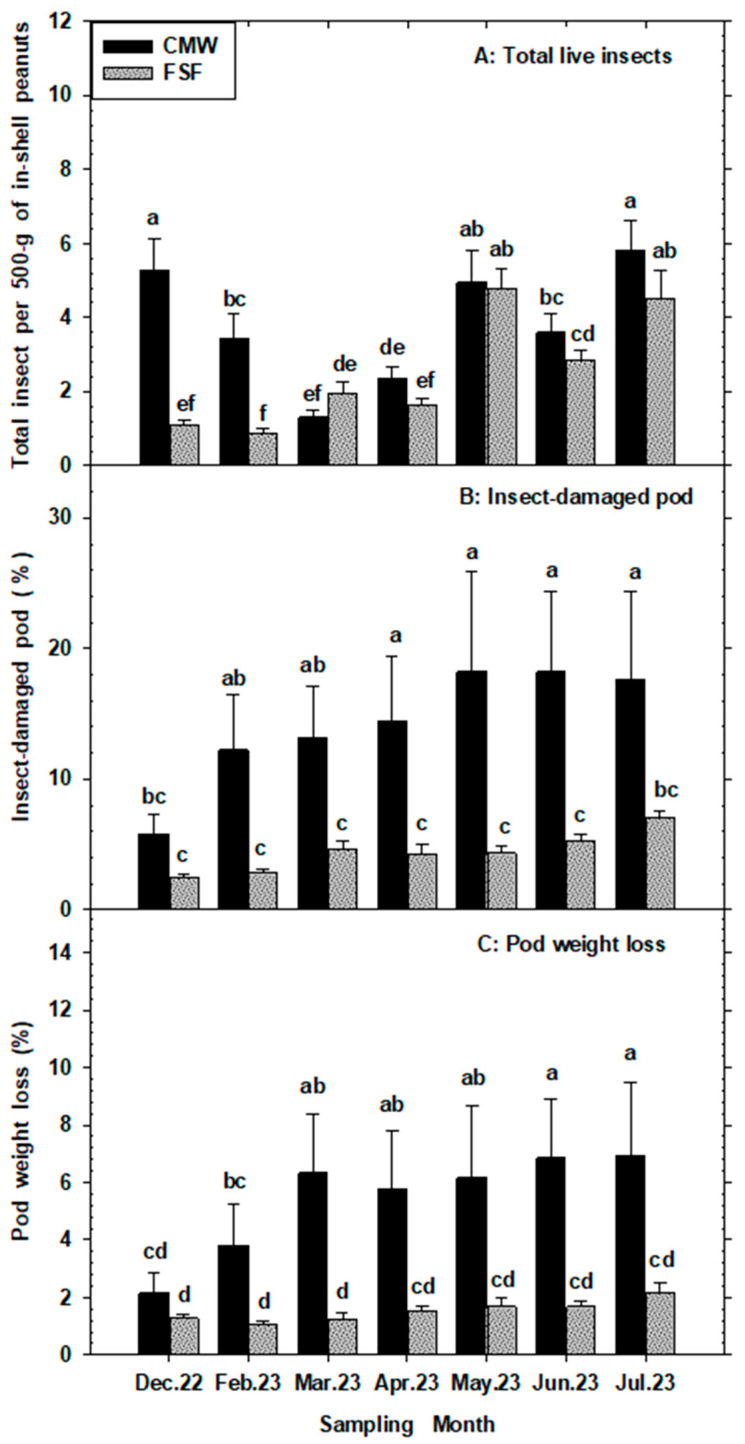
Mean (±SE) numbers of live stored product insect pests, insect-damaged pod, and pod weight loss in a 500 g sample of in-shelled peanuts from commercial warehouses—conventional metal warehouses (CMWs) and flat storage facilities (FSFs)—in Georgia, USA sampled during 2022–2023 storage period. For each variable, significant differences between CMWs and FSFs are denoted by different lower-case letters, (*p* < 0.05, LSMeans under Proc GLIMMIX in SAS).

The temporal abundance of stored-product insect pests varied significantly with both the month and the type of storage structure; however, the species diversity in both types of storage structures was similar (Table 2 and Table 3). The main effects of the month, storage structure, and interactions were significantly different (*p* < 0.05) for the total live insect species per 500 g of in-shell peanuts (Table 2, Figure 2A). Typically, more live insect pests were detected in peanut samples collected from conventional metal warehouses in December 2022 and February 2023 than in flat storage facilities. However, the levels of insect infestations were statistically similar in both types of storage structures in the subsequent months (Figure 2A). The relative abundance of identified stored-product insect pests (total captured = 18,161) included the order Lepidoptera (16.05%) [comprising *P. interpunctella* (6.65%), *E. kuehniella* (2.02%), *C. cautella* (4.90%), and *E. elutella* (2.48%)], Coleoptera (29.60%) [comprising *T. castaneum* (7.16%), *T. confusum* (2.47%), *C. pusillus* (2.46%), *Cryptolestes ferrugineus* Stephens (7.38%), *A. advena* (2.80%), *C. dimidiatus* (2.17%), *L. serricorne* (0.93%), *Stegobium paniceum* (Linnaeus) (0.78%), *T. variabile* (1.14%), *T. stercorea* (1.51%), *Rhyzopertha dominica* Fabricius (0.75%), and *Tenebrio molitor* Linnaeus (0.03%)], and Psocodea (54.35%) [comprising *Liposcelis entomophila* (Enderlein) (23.28%), *Liposcelis decolor* (Pearman) (14.18%), *Liposcelis bostrychophila* (Badonnel) (10.88%), and *Lachesilla pedicularia* (Linnaeus) (6.02%)]. Moreover, predatory arthropods, *Cheyletus* spp. (total captured = 1003) and *Xylocoris flavipes* (Reuter) (total captured = 266) were recovered from peanut samples.

Separate analyses were performed to determine the fixed effects of month and storage structure, along with their interactions, on *P. interpunctella*, *C. cautella*, *E. kuehniella*, *E. elutella*, *T. castaneum*, *T. confusum*, *C. ferrugineus*, *A. advena*, *C. dimidiatus*, *T. stercorea*, *L. entomophila*, *L. decolor*, *L. bostrychophila*, and *L. pedicularia*. For the overall analyses, the main effects of month and storage structure, and the interaction, were significant (*p* < 0.05) for all species except *E. elutella*, *E. kuehniella*, *A. advena*, *T. stercorea*, and *L. bostrychophila* (Table 2). The main effects of month and storage structure were significant at *p* < 0.05 for *A. advena* and *T. stercorea*; however, only the main effect of storage structure significantly (*p* < 0.05) influenced the *L. bostrychophila* population dynamics. However, the month, storage structure, and interactions were not significant (*p* > 0.05) for *E. elutella* and *E. kuehniella* (Table 2). High numbers of *P. interpunctella* were found in peanuts collected from conventional metal warehouses (CMWs) compared to flat storage facilities (FSFs). The mean *P. interpunctella* infestation levels ranged from 1.2 to 8.3 moths per 500 g of in-shell peanuts in CMWs, and from 1.1 to 4.6 moths per 500 g of in-shell peanuts in FSFs. The peak population levels of *P. interpunctella* occurred in December and March in CMWs, and in March, May, and June in FSFs (Table 3). Significantly higher levels of *C. cautella* were collected from CMWs, with a mean of 2.89 ± 0.94 moths per 500 g of in-shell peanuts, compared to FSFs, which had a mean of 2.5 ± 0.58 moths per 500 g of in-shell peanuts. Also, maximum infestation levels of *C. cautella* were observed in December and June in CMW samples, and in May and June in FSF samples. Regarding *E. kuehniella*, sample populations ranged from 0.8 to 2.3 moths per 500 g of peanuts in CMWs and from 0.4 to 1.5 moths per 500 g of peanuts in FSFs. Similarly, *E. elutella* sample populations ranged from 0.7 to 1.9 moths per 500 g of in-shell peanuts in CMWs and from 0.3 to 2.2 moths per 500 g in FSF peanut samples (Table 3).

Regarding infestation by beetles, the mean number of *T. castaneum* was significantly higher in samples from the conventional metal warehouses compared to samples from the flat storage facilities, specifically during the months of February, May, June, and July. The peak population of *T. castaneum* was observed in May and June, with densities exceeding 17.3 beetles per 500 g of peanuts. No infestations were detected in FSF samples during the winter months of December and February (Table 3). Similarly, the population of *T. confusum* was considerably higher in the CMWs compared to the FSFs during May, June, and July. However, no significant differences were observed in *T. confusum* populations between the storage structures or among the months from December through April. *Cryptolestes ferrugineus* was the most predominant beetle recovered from the peanuts, with the highest infestation levels observed in the CMWs during the late storage months of May and July (22.0 to 26.8 beetles per 500 g peanuts) (Table 3). Additionally, the population dynamics of *A. advena* followed a similar trend to that of *C. ferrugineus* in terms of infestation patterns across storage structures and over different months. The population of *C. dimidiatus* was significantly high in the CMWs in December (4.1 ± 2.0 beetles) and in both storage structures in July (≥3.5 beetles) (Table 3). Moreover, there was no significant infestation of *T. stercorea* observed in either storage structure (mean infestation ≤ 1.1 beetles) until May when the highest number of this insect (3.1 ± 1.6 beetles) was recorded for CMW samples (Table 3).

The order Psocodea had the highest number of individuals in samples from the different types of storage structures monitored. *Liposcelis entomophila* numbers were considerably high in the initial storage months (December and February) in CMWs (≥25.2 psocids), followed by a decline until June, and then a significant rebound in July. Conversely, in FSFs, the peak population of *L. entomophila* was observed in July (35.3 psocids), representing approximately a 27.2-fold increase compared to the initial sampling month (1.3 psocids) (Table 3). Additionally, *L. decolor* populations were significantly high in December (20.8 psocids) and July (13.8 psocids) in CMWs, and in May (17.3 psocids) for FSFs. For the remaining months, the mean number of *L. decolor* recovered in CMWs and FSFs was similar (≤8.5 psocids) (Table 3). In the case of *L. bostrychophila*, infestation levels remained consistently similar across months in CMWs (≤12.3 psocids), while significantly higher levels were detected in May (18.9 psocids) in FSF samples. *Lachesilla pedicularia* populations were significantly high in December (≥6.8 psocids) in both types of storage structures; however, no *L. pedicularia* were recovered from peanuts sampled during the spring and summer months (March–July) in CMWs or during the summer months (June and July) in FSFs (Table 3).

A statistically significant positive correlation was observed between the ambient temperature in storage structures and the total number of insects recovered (r = 0.82, *p* = 0.021), and between the equilibrium moisture content (EMC) of peanuts and the total number of insects (r = 0.81, *p* = 0.024). However, there was no significant correlation between ambient relative humidity (r.h.) and the total number of insects detected (r = −0.05, *p* = 0.905). When evaluating the insect species separately, the association of *T. castaneum* (r = 0.86), *T. confusum* (r = 0.94), *C. ferrugineus* (r = 0.93), *A. advena* (r = 0.92), *C. dimidiatus* (r = 0.93), *T. stercorea* (r = 0.97), and *L. entomophila* (r = 0.79) with temperature were significant (*p* < 0.05). Also, among all insect species recovered, only *L. pedicularia* had a significant correlation with r.h. (r = 0.78, *p* = 0. 038). Moreover, the correlation of *C. cautella* (r = 0.79), *E. elutella* (r = 0.75), *T. castaneum* (r = 0.84), *T. confusum* (r = 0.89), *C. ferrugineus* (r = 0.85), *A. advena* (r = 0.81), *C. dimidiatus* (r = 0.81), and *T. stercorea* (r = 0.90) with the EMC of peanuts were all significant (*p* < 0.05).

### 3.3. Percentage Insect-Damaged Pods (%IDP), Pod Weight Loss (%PWL), and Cracked Pod (%CP) per 500 g of Peanuts

**Figure 3:**
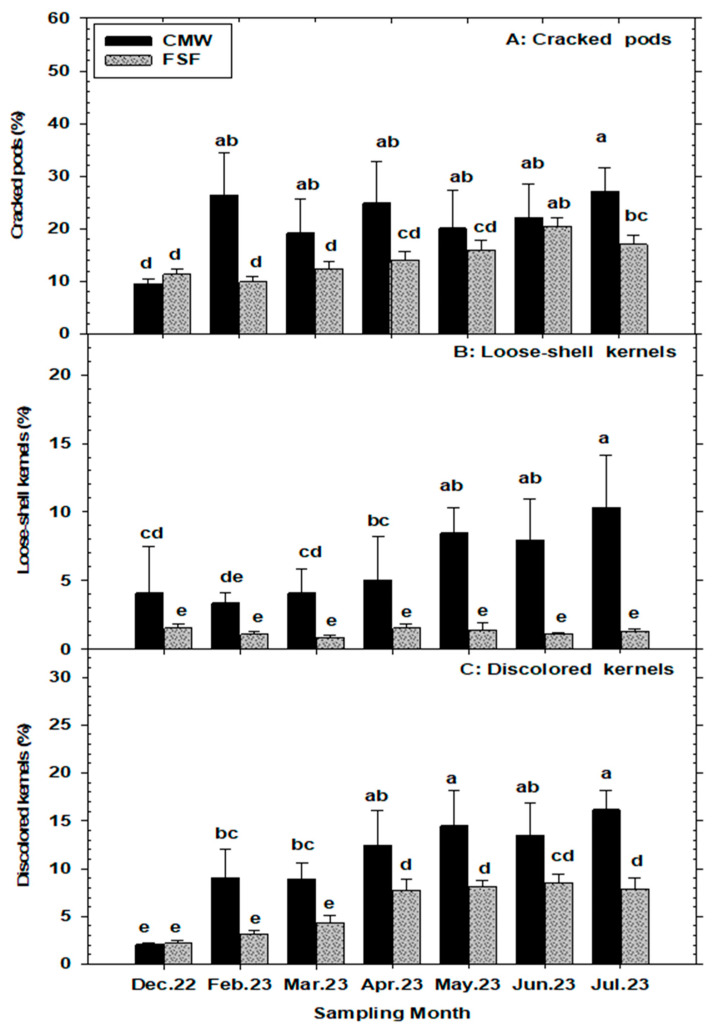
Mean (±SE) numbers of cracked pods, loose-shell kernels, and discolored kernels in a 500 g sample of in-shelled peanuts from commercial warehouses—conventional metal warehouses (CMWs) and flat storage facilities (FSFs)—in Georgia, USA sampled during 2022–2023 storage period. For each variable, significant differences between CMWs and FSFs are denoted by different lower-case letters, (*p* < 0.05, LSMeans under Proc GLIMMIX in SAS).

The primary effects of month and storage structure were statistically significant (*p* < 0.05) for all three peanut pods soundness variables (%IDP, %PWL, and %CP). However, the interaction between these main effects was not significant (*p* > 0.05) (Table 1). Percent insect-damaged pods (%IDP) and pod weight loss (%PWL) were consistently and substantially higher in the CMWs (conventional metal warehouses) compared to the FSFs (flat storage facilities) across most months, except in December. Additionally, %IDP and %PWL remained stable in the FSFs across all months, while in the CMWs, both variables were lowest at the beginning of the storage period and increased significantly over time. The monthly averages of %IDP in CMWs and FSFs ranged from 5.8% to 18.2% and 2.4% to 7.0%, respectively. Similarly, %PWL ranged from 2.2% to 6.9% in CMWs and from 1.0% to 2.1% in FSFs (Figure 2B,C). Furthermore, the percentage of cracked pods (%CP) was mostly higher in the CMWs than in the FSFs across most months, except in December and June. In both storage structures, %CP increased significantly over the storage period, with monthly means ranging from 9.5% to 26.3% in CMWs and from 9.9% to 20.4% in FSFs (Figure 3A).

The levels of damaged-pod variables of peanuts (%IDP, %PWL, and %CP) showed positive associations with ambient storage temperature and equilibrium moisture content (EMC) of peanuts, but not with ambient storage relative humidity (r.h.). Percent IDP increased with temperature (r = 0.91, *p* = 0.004) and EMC (r = 0.80, *p* = 0.028), but showed no significant correlation with r.h. (r = −0.37, *p* = 0.405). Additionally, %IDP significantly (*p* < 0.05) increased or decreased with infestations of *C. cautella* (r = 0.78), *T. confusum* (r = 0.83), *C. ferrugineus* (r = 0.83), *C. dimidiatus* (r = 0.87), *T. stercorea* (r = 0.83), *L. entomophila* (r = 0.80), and *L. pedicularia* (r = −0.79) during peanut storage. Similarly, %PWL increased with temperature (r = 0.92, *p* = 0.003) and EMC (r = 0.92, *p* = 0.002), but had no significant association with r.h. (r = −0.07, *p* = 0.880). Percent PWL also significantly (*p* < 0.05) increased with the total number of insects recovered from peanut samples (r = 0.83), *T. castaneum* (r = 0.78), *T. confusum* (r = 0.90), *C. ferrugineus* (r = 0.94), *A. advena* (r = 0.85), *C. dimidiatus* (r = 0.89), *T. stercorea* (r = 0.92), and *L. entomophila* (r = 0.82). Moreover, %CP had a significantly strong positive relationship with storage temperature (r = 0.86, *p* = 0.011) and EMC (r = 0.87, *p* = 0.009), but no significant association with r.h. (r = −0.09, *p* = 0.837). Furthermore, %CP exhibited a significant (*p* < 0.05) positive association with the numbers of *C. cautella* (r = 0.84), *E. elutella* (r = 0.89), *T. castaneum* (r = 0.90), *T. confusum* (r = 0.89), *A. advena* (r = 0.82), and *T. stercorea* (r = 0.90).

### 3.4. Percentage Insect-Damaged Kernels (%IDK), Kernel Weight Loss (%KWL), Loose-Shelled Kernel (%LSK), and Discolored Kernel (%DK) per 500 g of Peanuts

**Figure 4:**
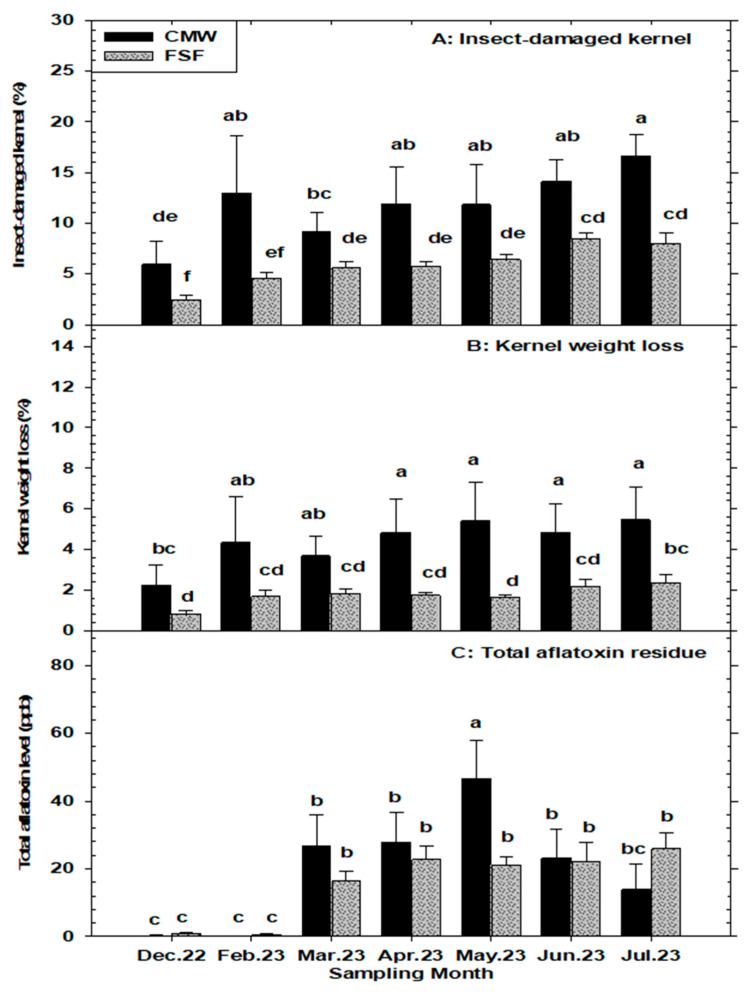
Mean (±SE) numbers of insect-damaged kernels, kernel weight loss, and total aflatoxin levels in a 500 g sample of in-shelled peanuts from commercial warehouses—conventional metal warehouses (CMWs) and flat storage facilities (FSFs)—in Georgia, USA sampled during 2022–2023 storage period. For each variable, significant differences between CMWs and FSFs are denoted by different lower-case letters, (*p* < 0.05, LSMeans under Proc GLIMMIX in SAS).

The main effects of month and storage structure were statistically significant (*p* < 0.05) for the percentage of insect-damaged kernels (%IDK), percentage kernel weight loss (%KWL), and percent discolored kernels (%DK). However, the interaction effects on these variables were not significant (*p* > 0.05). In contrast, the effects of the month, storage structure, and their interactions were all significant (*p* < 0.05) for percentage loose-shelled kernels (%LSK) (Table 1). The percentage of insect-damaged kernels (%IDK) was significantly higher in samples from conventional metal warehouses compared to flat storage facilities, ranging from 5.9% to 16.6% and 2.4% to 8.0%, respectively. A trend of increasing %IDK with storage duration was observed in both storage structures, with the lowest and highest %IDKs recorded in December and July, respectively (Figure 4A). Percentage kernel weight loss (%KWL) due to insect infestations followed a similar pattern to %IDK, with monthly averages in CMWs and FSFs ranging from 2.2% to 5.4% and 0.82% to 2.3%, respectively (Figure 4B). Similarly, the percentage of discolored kernels (%DK) was generally higher in peanut samples from CMWs compared to FSFs across all months, except in the initial sampling month (December). The %DKs in CMWs and FSFs ranged from 2.0% to 16.1% and 2.1% to 7.8%, respectively (Figure 3C). Furthermore, the percentage of loose-shelled kernels (%LSK) was consistently and significantly greater in samples from the CMWs across months compared to the FSFs. In both storage systems, %LSK fluctuated throughout the sampling months, with monthly averages ranging from 3.3% to 10.3% in CMWs and from 0.88% to 1.5% in FSFs (Figure 3B).

For damaged-kernel variables in stored peanuts, the percentage of insect-damaged kernels (%IDK) showed a significantly strong positive relationship with temperature (r = 0.92, *p* = 0.003), but exhibited no significant association with relative humidity (r = −0.46, *p* = 0.295) and equilibrium moisture content of peanuts (r = 0.71, *p* = 0.069). Similarly, kernel weight loss (%KWL) due to insect infestations increased significantly with an increase in storage temperature (r = 0.83, *p* = 0.019), but had no significant association with r.h. (r = −0.60, *p* = 0.150) or EMC (r = 0.51, *p* = 0.237). For loose-shell kernels (%LSK), there were no significant associations with temperature (r = −0.30, *p* = 0.939), r.h. (r = 0.35, *p* = 0.434), or EMC (r = 0.20, *p* = 0.652). Increasing levels of discolored kernels (%DK) in stored peanuts were significantly associated with increases in storage temperature (r = 0.83, *p* = 0.018) and EMC (r = 0.75, *p* = 0.048), but showed no significant relationship with r.h. (r = −0.44, *p* = 0.314). Additionally, %IDK in stored peanuts tended to show a significant (*p* < 0.05) association with the infestation by *C. cautella* (r = 0.84), *E. elutella* (r = 0.80), *T. castaneum* (r = 0.84), *T. confusum* (r = 0.88), *C. ferrugineus* (r = 0.77), *A. advena* (r = 0.80), *C. dimidiatus* (r = 0.75), *T. stercorea* (r = 0.87), and *L. pedicularia* (r = −0.89). Similarly, %DK exhibited a significant (*p* < 0.05) association with *C. cautella* (r = 0.01), *E. elutella* (r = 0.92), *T. castaneum* (r = 0.84), *T. confusum* (r = 0.91), *C. ferrugineus* (r = 0.79), *A. advena* (r = 0.78), *T. stercorea* (r = 0.86), and *L. pedicularia* (r = −0.73). Only *L. pedicularia* had a significant association (r = −0.93, *p* = 0.001) with %KWL for all the insect species recovered. There was no significant (*p* > 0.05) relationship between %LSK and any of the insect species recovered from peanut samples.

### 3.5. Total Aflatoxin Levels (ppb) in Stored In-Shell Peanuts

**Figure 5:**
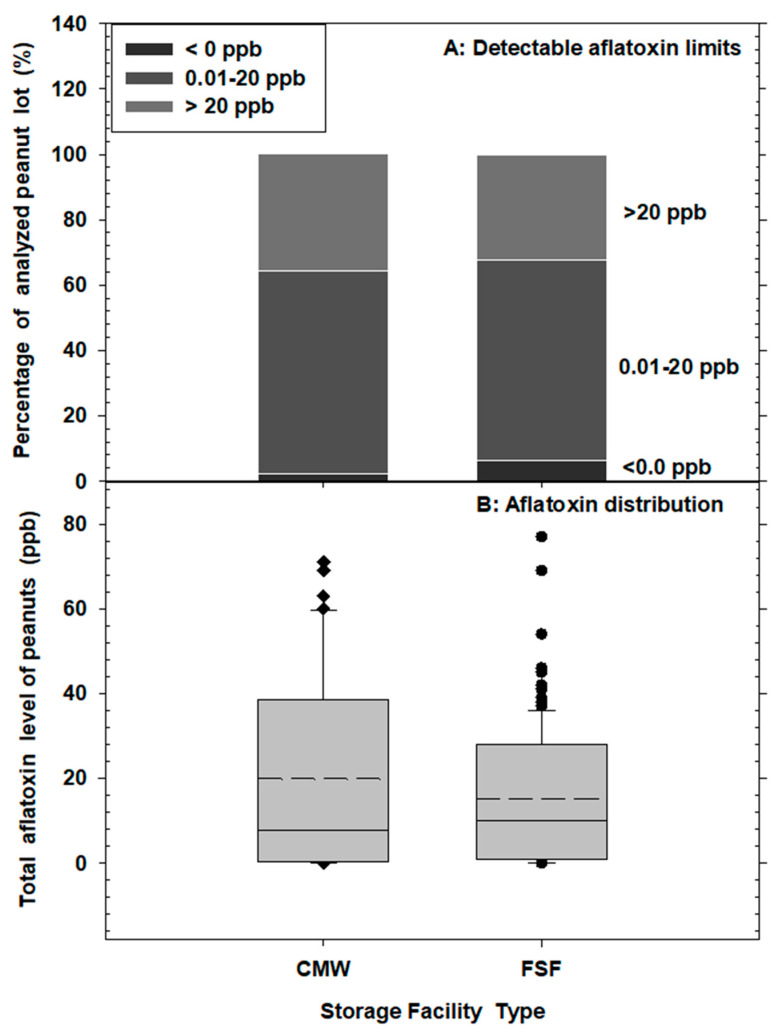
Percentage detectable aflatoxin limits and aflatoxin distribution of analyzed peanut lots sampled from commercial warehouses—conventional metal warehouses (CMWs) and flat storage facilities (FSFs)—in Georgia, USA during 2022–2023 storage period. Comparatively, the distribution of aflatoxin in CMWs and FSFs presented as box plots (short-dashes = mean aflatoxin residue) was not significantly different (DF 1,151 *F* = 2.06 *p* = 0.1531, under the Proc NPAR1WAY in SAS).

The interaction between the fixed effects of month and storage structure on total aflatoxin levels in peanut samples was statistically significant (*p* < 0.05) (Table 1). Monthly average aflatoxin concentrations were generally similar in both storage systems across sampling months, except in May, where significantly higher aflatoxin levels were detected in peanut samples stored in conventional metal warehouses (CMWs) compared to those in flat storage facilities (FSFs) (Figure 4C). In both storage structures, aflatoxin levels were significantly lower during the early storage period (winter months: December to February), but there was a notable increase in aflatoxin concentrations during the late storage period (spring and summer months: March to July). Specifically, there was an approximately 110.7-fold and 14.4-fold increase in aflatoxin concentrations in peanut samples from CMWs and FSFs, respectively. The average total aflatoxin residues ranged from 0.2 to 46.7 ppb in the CMWs and from 0.8 to 25.8 ppb in the FSFs across the sampling months (Figure 4C). Combined analysis of peanut lots showed that approximately 2.3%, 61.9%, and 35.7% of the CMW peanut lots had total aflatoxin levels of <0.0 ppb, 0.01–20 ppb, and >20 ppb, respectively. Similarly, 6.3%, 61.2%, and 32.4% of the FSF peanut lots had total aflatoxin levels of <0.0 ppb, 0.01–20 ppb, and >20 ppb, respectively (Figure 5A). Overall mean total aflatoxin levels were 19.8 ppb (range: 0.0–71 ppb) in CMWs and 15.0 ppb (range: 0.0–77 ppb) in FSFs (Figure 5B) in test samples.

A statistically significant positive correlation was observed between the ambient temperature in storage structures and the total aflatoxin levels in peanuts (r = 0.80, *p* = 0.028), as well as between the equilibrium moisture content (EMC) of peanuts and the total aflatoxin levels (r = 0.79, *p* = 0.03). However, there was no significant correlation between ambient relative humidity (r.h.) and the total aflatoxin residues in peanuts (r = −0.52, *p* = 0.225). Similarly, total aflatoxin levels showed no significant correlation with the total number of insects recovered from peanut samples (r = 0.73, *p* = 0.057). However, when the insect species were evaluated separately, the association of *P. interpunctella* (r = 0.75), *C. cautella* (r = 0.91), *E. kuehniella* (r = 0.76), *E. elutella* (r = 0.86), *T. castaneum* (r = 0.78), *T. confusum* (r = 0.86), *C. ferrugineus* (r = 0.76), *T. stercorea* (r = 0.80), and *L. pedicularia* (r = −0.79) infestations with aflatoxin residues in peanut samples were all significant (*p* < 0.05). Regarding peanut soundness variables, only %IDP (r = 0.86), %PWL (r = 0.81), %CP (r = 0.78), %IDK (r = 0.85), and %DK (r = 0.92) showed significant (*p* < 0.05) correlation with increasing levels of aflatoxin contamination in stored peanuts.

## 4. Discussion

This study highlights the significant impact of storage systems and physical environmental variables on the quality and safety of bulk-stored peanuts in the southeastern United States. Results indicate that conventional metal warehouses generally maintain higher ambient temperatures (18.8 to 32.6 °C) compared to flat storage facilities (15.8 to 30.6 °C), with both types of structures sustaining similar temperatures (~13.6 °C) during the early winter season (December). The higher temperatures in conventional metal warehouses are likely due to their structural composition and design, which include steel frames, walls, roofs, and doors that facilitate rapid heating by external weather conditions. In contrast, flat storage facilities often utilize a combination of steel and wood, sometimes with insulation [18]. The recorded temperature differential is crucial as it influenced the equilibrium moisture content (EMC) of peanuts, with samples from conventional metal warehouses showing a broader range of EMC (6.1 to 8.9%) compared to those from flat storage facilities (7.2 to 8.8%). Conversely, relative humidity (r.h.) showed greater fluctuations in flat storage facilities than in conventional metal warehouses, with r.h. gradients of 10.4% (ranging from 61.8 to 72.2%) and 7.1% (ranging from 54.9 to 62.0%), respectively. The variation and magnitude of these ambient environmental conditions, coupled with increased kernel moisture content—especially during the spring and summer months (March through July)—likely facilitated the proliferation of stored-product insect pests, mold growth, and aflatoxin production in stored peanuts. Previous studies have shown that elevated temperatures, r.h., and equilibrium moisture content of grains and kernels in storage environments correlate with increased insect pests and microbial contamination, leading to significant quality degradation and economic losses in stored products [17,22,23,24,25,26]. Furthermore, the detection of *Ahasverus advena*, *Carpophilus dimidiatus*, *Typhaea stercorea*, and *Liposcelis* spp. indicates damp, moldy conditions in the sampled warehouses. These findings underscore the critical need for advanced temperature and humidity control systems in bulk storage facilities to enhance the long-term quality of farmer stock peanuts. Similarly, strategies such as routine turning of piled peanuts and the adoption of advanced ambient aeration or ventilation systems can effectively mitigate the accumulation of heat and moisture within peanut piles and prevent headspace and downspout condensation in farmer stock storage structures [4,27]. Additionally, the use of monolithic warehouses—constructed with reinforced concrete and concrete slabs—provides high structural integrity and significantly reduces fluctuations in ambient storage conditions caused by external weather factors [18]. They offer a promising alternative for long-term bulk storage of in-shell peanuts.

The market value of peanuts is intricately linked to their quality, with moisture content being a critical determinant. According to [18], the average moisture content of farmer stock peanuts is typically 8–9% at the time of grading. Under normal weather conditions, this moisture content will decrease to an equilibrium level of 5–7% as the peanuts lose moisture and heat. To prevent moisture reabsorption, it is essential that the evaporated moisture is effectively removed from the storage warehouse without condensing back onto the peanuts. Our results showed seasonal fluctuations in peanut moisture content, with consistently higher levels observed from late spring through the summer storage season for peanuts in flat storage facilities compared to conventional metal warehouses. In July, stored peanuts in both storage systems reached the highest equilibrium moisture content (EMC) during the storage period, with monthly averages of 8.8% and 8.9% in flat storage facilities and conventional metal warehouses, respectively. The peak EMC during the summer months (approximately 7.8% to 8.9%) strongly correlated with elevated ambient storage temperatures, ranging from 26.7 °C to 32.6 °C—conditions conducive to the growth, development, and survival of stored-product insect pests [3,14,28]. This rise in EMC, indicating the rehydration of in-shell peanuts, was also associated with a decline in peanut quality, evidenced by an increased percentage of discolored kernels and higher levels of aflatoxin contamination. These findings highlight the critical need for regular monitoring of moisture content in stored peanuts, particularly in the humid-subtropical climatic conditions present in the studied areas. High equilibrium moisture content can promote fungal and insect growth, as well as increase kernel respiration and rehydration through condensation within poor aerated storage systems [18,29,30]. Consequently, it is essential to install properly designed and operated warehouse ventilation and/or aeration systems in farmer stock storage structures to maintain the moisture content of in-shell peanuts at levels that inhibit insect pest proliferation and mold growth. Targeting kernel moisture content levels below 7% can help ensure the quality and stability of in-shell peanuts during long-term storage in farmer stock warehouses [31]. Similarly, routine inspections for leaks in roofs, walls, doors, and ventilators during rainy periods, along with regular checks for condensation, would be recommended.

Stored-product insect pests significantly impacted peanut quality in both conventional metal warehouses and flat storage facilities, with the extent of infestation varying by storage structure, storage season, storage duration, and insect species. Peanuts stored in conventional metal warehouses experienced approximately 1.5 times more insect infestations than those in flat storage facilities, with infestations greatest in late storage periods (late spring and summer months) and lowest in the early storage period (winter months). Early-storage infestations in farmer stock warehouses are linked to preharvest infestations, residual insect populations from previously stored consignments, and migrating external populations to warehouses [6,21,32]. The study identified *Psocodea* as the most abundant group infesting stored in-shell peanuts, while *Lepidoptera* and *Coleoptera* showed greater species diversity. Although species richness was similar across warehouses, *Plodia interpunctella*, *Cadra cautella*, *Ephestia kuehniella*, *Ephestia elutella*, *Tribolium castaneum*, *Tribolium confusum*, *Cryptolestes ferrugineus*, *Ahasverus advena*, *Carpophilus dimidiatus*, *Typhaea stercorea*, and *Liposcelis entomophila* were predominant in conventional metal warehouses, while *Liposcelis decolor*, *Liposcelis bostrychophila*, and *Lachesilla pedicularia* were more prominent in flat storage facilities. Interestingly, the predominant insect pests recovered from both storage structures were primarily external grain feeders that typically target farmer stock peanuts with high levels of foreign material and broken or damaged pods and kernels [23,33,34]. Moreover, the presence of these insects may indicate problems with sanitation programs in farmer stock warehouses [8,35].

Insect pest management in farmer stock warehouses typically targets primary insect pests, particularly moths, through scheduled fumigation. However, the role of externally feeding insect species in peanut storage is crucial due to their contribution to peanut quality deterioration and aflatoxin contamination, as shown in the current study. Species such as *P. interpunctella* and *T. castaneum*, along with *Liposcelis* spp., can damage peanut pods or kernels, creating entry points for mycotoxigenic fungi such as *Aspergillus flavus*, which leads to the production of aflatoxins [25]. Additionally, the activities of these insect pests have been reported to increase moisture and create localized microenvironments (pockets) within grain piles that are conducive to mold growth [3,23,33]. While *T. stercorea* and *Liposcelis* spp., detected in the current study, are mostly regarded as mycophagous, they can also transport viable aflatoxigenic inocula, further increasing the risks of aflatoxin contamination in stored peanuts. Given their high reproductive rates, the presence of *C. ferrugineus*, *T. castaneum*, and *L. entomophila* in peanut piles can disrupt storage conditions by raising temperature and relative humidity, creating a favorable environment for the proliferation of other pestiferous arthropods and microbial growth [36,37]. The prevalence of *T. castaneum*, *T. confusum*, *C. ferrugineus*, and *L. entomophila* showed strong positive correlations with temperature and equilibrium moisture content and their recovery rate remained substantially high 4–8 weeks after phosphine fumigation, especially in conventional metal warehouses. Calendar scheduled interventions, pesticide leakages in warehouses, and indiscriminatory exposure of different insect species to similar phosphine concentrations over time were reported to cause resistance buildup in populations of *T. castaneum* and *C. ferrugineus* [38,39,40]. Moreover, *Liposcelis* spp. including *L. entomophila,* the predominant insect infesting peanuts in the studied areas were shown to be naturally tolerant to phosphine [41,42]. This situation, coupled with the detection of species of phytosanitary interest, including *Lasioderma serricorne*, *Stegobium paniceum*, and *Rhyzopertha dominica*, poses significant economic risks and challenges to the USA peanut industry in meeting local and international regulatory standards for peanut marketability. For instance, the USDA-FSIS and the European Commission impose strict limits on live insect presence in stored peanuts, often specifying a zero-tolerance policy [43,44]. Moreover, the regulatory threshold for dead insects in stored commodities is set at 75 fragments per 50 g sample. Exceeding these thresholds can lead to the rejection or downgrading of peanuts and market access issues. These challenges emphasize the critical need for tailored insect pest management programs, particularly in conventional metal warehouses and in summer storage seasons where higher temperatures may favor insect proliferation. Additionally, the presence of diverse insect species across multiple insect orders and families underscores the complexity of implementing effective ecological, economical, and sustainable integrated pest management (IPM) programs to ensure the minimization of insect pest-related losses and the safety of stored peanuts. These programs must be carefully designed to address the specific challenges posed by each storage structure, incorporate regular insect monitoring plans, and establish thresholds for insect pest management decisions.

The percentage of insect-damaged pods (IDP), pod weight loss (PWL), and cracked pods (CP) showed positive correlations with total insect pests captured, temperature, and equilibrium moisture content (EMC), but not with relative humidity, with these damage indicators consistently higher in conventional metal warehouses compared to flat storage facilities. The quality of peanut kernels was significantly affected by these ecological variables, resulting in higher percentages of insect-damaged kernels (IDK), kernel weight loss (KWL), and discolored kernels (DK) in conventional metal warehouses. Interestingly, the percentage of loose-shelled kernels (LSKs) did not show a significant positive correlation with any of the ecological variables examined, yet it remained consistently higher in conventional metal warehouses. Insect-damaged kernels ranged from 5.9% to 16.6% in conventional metal warehouses compared to 2.4% to 8.0% in flat storage facilities, both showing a similar increasing trend over time. Kernel weight loss varied from 2.2% to 5.4% in conventional metal warehouses and from 0.82% to 2.3% in flat storage facilities, while DKs ranged from 2.0% to 16.1% in conventional metal warehouses and from 2.1% to 7.8% in flat storage facilities. Prior to harvesting and transportation from growers’ fields to buying points, preharvest quality losses of peanuts in principal production areas of Georgia were reported by [21], with quality parameters significantly lower than those found at the storage stage. High infestations of in-shell peanuts by moths, such as *Cadra cautella* and *Ephestia elutella*, were significantly associated with higher percentages of insect-damaged pods, cracked pods, insect-damaged kernels, discolored kernels, and pod weight loss. Similarly, increased infestations of beetles, particularly *T. castaneum*, *T. confusum*, *C. ferrugineus*, *A. advena*, *C. dimidiatus*, and *T. stercorea*, correlated with elevated levels of insect-damaged pods, cracked pods, pod weight loss, insect-damaged kernels, and discolored kernels. Despite the high infestation of psocids in warehouses, only *L. entomophila* significantly correlated with increased levels of peanut pod quality variables deterioration over the storage period.

High levels of insect-damaged kernels and loose-shelled kernels are major grading components of peanuts that attract substantial penalties and economic losses in the peanut industry because they are more likely to be contaminated with aflatoxin [43]. Thus, IDKs and LSKs are critical grading parameters, with USDA-FSIS standards allowing no more than 3.49% damaged kernels and 5.0% LSKs for premium grades. While most samples from flat storage facilities were within the acceptable threshold, the majority of peanut samples from the conventional metal warehouses exceeded the limit, particularly samples from the summer storage months, which could impact their suitability for export to stringent markets such as the EU. Exceeding regulatory limits for percentage insect-damaged kernels and loose-shelled kernels can lead to increased processing costs and reduced quality, affecting the overall profitability of peanuts [2,45]. However, for most domestic markets and certain uses (e.g., the oil market), these levels are still considered acceptable under USDA-FSIS regulations [43].

Aflatoxin concentrations were low during the winter months (early storage period) but increased by at least 110.7-fold and 14.4-fold during spring and summer (late storage period) in samples from conventional metal warehouses and flat storage facilities, respectively. Monthly averages ranged from 0.2 to 46.7 ppb in conventional metal warehouses and 0.8 to 25.8 ppb in flat storage facilities, with an overall average of 16.37 ppb across the studied peanut lots. Preharvest aflatoxin values in Georgia, USA averaged 0.59 ppb, with a range of 0.028 to 4.3 ppb [21], highlighting a 27.74-fold increase in aflatoxin risk during postharvest storage of peanuts. The FDA in the USA sets regulatory limits for total aflatoxin (B_1_, G_1_, B_2_, and G_2_) in peanuts and peanut products at 20 ppb for human consumption, except raw peanuts that will be further processed to remove moldy and otherwise defective nuts, while the European Union imposes a stricter limit of 4 ppb [44,46]. Exceeding these thresholds poses significant health risks and economic losses due to rejected shipments and reduced market access. The study also revealed a strong correlation between total aflatoxin residues and both ambient temperature and equilibrium moisture content of peanuts, but not with ambient relative humidity. A similar trend was reported by [23] and [9] in corn stored in ZeroFly storage bags, cribs, steel bins, and concrete warehouses under tropical conditions. Moreover, increased aflatoxin contamination was directly linked to infestations by various stored-product insect pests, including *P. interpunctella*, *C. cautella*, *E. kuehniella*, *E. elutella*, *T. castaneum*, *T. confusum*, *C. ferrugineus*, and *T. stercorea*, underscoring the importance of effective insect pest control strategies in managing aflatoxin levels in stored peanuts. Furthermore, high levels of insect-damaged pods, pod weight loss due to insect feeding activities, cracked pods, insect-damaged kernels, and discolored kernels in farmer stock peanuts, were also predictive of elevated aflatoxin contamination during storage, emphasizing the need for improved storage conditions and integrated management practices to mitigate these risks.

## 5. Conclusions

The current study highlights the significant influence of storage systems, insect pest infestations, and environmental conditions on aflatoxin contamination and other peanut quality variables in the southeastern United States. Peanuts stored in conventional metal warehouses experienced higher ambient temperatures, moisture content, and insect infestations, leading to greater aflatoxin contamination and peanut quality deterioration compared to those stored in flat storage facilities. Although species diversity was similar across warehouses, *P. interpunctella*, *C. cautella*, *E. kuehniella*, *E. elutella*, *T. castaneum*, *T. confusum*, *C. ferrugineus*, *A. advena*, *C. dimidiatus*, *T. stercorea*, and *L. entomophila* were predominant in conventional metal warehouses, while *L. decolor*, *L. bostrychophila*, and *L. pedicularia* were more prominent in flat storage facilities. High infestations of stored peanuts by moths, such as *Cadra cautella* and *Ephestia elutella*, were significantly associated with higher percentages of insect-damaged pods, cracked pods, insect-damaged kernels, discolored kernels, and pod weight loss. Similarly, increased infestations of beetles, particularly *T. castaneum*, *T. confusum*, *C. ferrugineus*, *A. advena*, *C. dimidiatus*, and *T. stercorea*, correlated with elevated levels of insect-damaged pods, cracked pods, pod weight loss, insect-damaged kernels, and discolored kernels. In both storage structures, aflatoxin levels were significantly lower during the early storage period (December to February), but there was a notable increase in aflatoxin concentrations during the late storage period (March to July). That is, there was an approximately 110.7-fold and 14.4-fold increase in aflatoxin concentrations in peanut samples from CMWs and FSFs, respectively. Given the substantial economic and health risks posed by aflatoxin contamination in postharvest peanuts, it is crucial to implement more effective integrated pest management strategies in farmer stock warehouses, particularly focusing on developing advanced storage technologies that maintain optimal environmental conditions for long-term storage of peanuts. Future research should explore the effectiveness of advanced storage systems, such as monolithic warehouses for sustaining long-term peanut quality during storage. Additionally, investigating the resistance mechanisms of stored-product insect pests to fumigants, particularly, phosphine, alongside the efficacy of alternative eco-friendly control measures, particularly biocontrol agents that were detected in the sampled storage structures (*Cheyletus* spp. and *Xylocoris flavipes*) and biopesticides (entomopathogenic nematodes and fungi), could provide valuable insights into enhancing integrated pest management practices in peanut storage.

## Figures and Tables

**Table 1:** Summary of tests of fixed effects of sampling month (M) and storage structure (ST), and interactions (*) for peanut physical condition (soundness and purity) parameters and ambient storage environmental conditions in farmer stock peanuts stored in commercial warehouses in Georgia, USA.

Variable	Source	DF	*F* Value	*p* Value
Kernel moisture content (%MC)	M	6,292	12.19	<0.0001
	ST	1,292	23.56	<0.0001
	M * ST	6,292	1.86	0.0869
Ambient temperature (T°C)	M	6,210	74.52	<0.0001
	ST	1,210	18.51	<0.0001
	M * ST	6,210	0.77	0.5926
Ambient relative humidity (% r.h.)	M	6,210	4.25	0.0005
	ST	1,210	68.04	<0.0001
	M * ST	6,210	0.84	0.5378
Insect-damaged pod (%IDP)	M	6,139	2.96	0.0095
	ST	1,139	62.94	<0.0001
	M * ST	6,139	1.11	0.3605
Pod weight loss (%PWL)	M	6,139	2.60	0.0203
	ST	1,139	68.04	<0.0001
	M * ST	6,139	1.52	0.1756
Cracked pod (%CP)	M	6,139	2.85	0.0120
	ST	1,139	15.98	0.0001
	M * ST	6,139	1.85	0.0930
Loose-shelled kernel (%LSK)	M	6,139	2.64	0.0185
	ST	1,139	61.41	<0.0001
	M * ST	6,139	2.22	0.0443
Discolored kernel (%DK)	M	6,139	11.73	<0.0001
	ST	1,139	39.01	<0.0001
	M * ST	6,139	1.43	0.2059
Insect-damaged kernel (%IDK)	M	6,139	5.21	<0.0001
	ST	1,139	47.59	<0.0001
	M * ST	6,139	0.80	0.5718
Kernel weight loss (%KWL)	M	6,139	2.18	0.0486
	ST	1,139	45.66	<0.0001
	M * ST	6,139	0.60	0.7327
Total aflatoxin level (ppb)	M	6,139	12.73	<0.0001
	ST	1,139	2.37	0.1262
	M * ST	6,139	2.55	0.0226

**Table 2:** Summary of tests of fixed effects of sampling month (M) and storage structure (ST), and interactions (*) for fourteen different stored-product insect species in farmer stock peanuts stored in commercial warehouses in Georgia, USA.

Variable	Source	DF	*F* Value	*p* Value
*Plodia interpunctella*	M	6,292	3.27	0.0040
	ST	1,292	8.11	0.0047
	M * ST	6,292	4.96	<0.0001
*Cadra cautella*	M	6,292	2.92	0.0088
	ST	1,292	0.70	0.4040
	M * ST	6,292	2.92	0.0088
*Ephestia kuehniella*	M	6,292	0.49	0.8151
	ST	1,292	0.08	0.7827
	M * ST	6,292	1.66	0.1294
*Ephestia elutella*	M	6,292	1.46	0.1906
	ST	1,292	0.03	0.8737
	M * ST	6,292	0.80	0.5702
*Tribolium castaneum*	M	6,292	8.38	<0.0001
	ST	1,292	37.25	<0.0001
	M * ST	6,292	2.54	0.0205
*Tribolium confusum*	M	6,292	12.54	<0.0001
	ST	1,292	47.29	<0.0001
	M * ST	6,292	6.07	<0.0001
*Cryptolestes ferrugineus*	M	6,292	7.58	<0.0001
	ST	1,292	35.38	<0.0001
	M * ST	6,292	3.76	0.0013
*Ahasverus advena*	M	6,292	5.90	<0.0001
	ST	1,292	13.21	0.0003
	M * ST	6,292	1.12	0.3527
*Carpophilus dimidiatus*	M	6,292	4.64	0.0002
	ST	1,292	6.46	0.0115
	M * ST	6,292	3.28	0.0039
*Typhaea stercorea*	M	6,292	6.27	<0.0001
	ST	1,292	4.66	0.0318
	M * ST	6,292	0.50	0.8065
*Liposcelis entomophila*	M	6,292	5.01	<0.0001
	ST	1,292	0.03	0.8638
	M * ST	6,292	6.60	<0.0001
*Liposcelis decolor*	M	6,292	3.51	0.0023
	ST	1,292	0.41	0.5223
	M * ST	6,292	2.68	0.0149
*Liposcelis bostrychophila*	M	6,292	3.81	0.0011
	ST	1,292	1.58	0.2092
	M * ST	6,292	0.89	0.5003
*Lachesilla pedicularia*	M	6,292	13.43	<0.0001
	ST	1,292	0.30	0.5837
	M * ST	6,292	2.69	0.0147
Total live insect spp.	M	6,5800	15.79	<0.0001
	ST	1,5800	24.36	<0.0001
	M * ST	6,5800	5.54	<0.0001

**Table 3:** Mean (±SE) numbers of stored-product insect species in a 500 g sample of in-shelled peanuts from commercial warehouses—conventional metal warehouses (CMWs) and flat storage facilities (FSFs)—in Georgia, USA sampled during 2022–2023 storage period. Differences between CMWs and FSFs for each insect species are denoted by lower-case letters (*p* < 0.05, LSMeans under Proc GLIMMIX in SAS).

Insect Species	Storage Structure			Sampling Month. Year				
		Dec.22	Feb.23	Mar.23	Apr.23	May.23	Jun.23	Jul.23
*P. interpunctella*	CMW	8.3 ± 1.8 a	3.6 ± 1.5 cd	5.6 ± 1.3 ab	5.2 ± 1.6 bc	2.3 ± 0.8 cd	5.3 ± 1.3 bc	1.2 ± 0.6 d
	FSF	1.7 ± 0.5 d	1.1 ± 0.3 d	3.9 ± 0.7 bc	2.9 ± 0.5 cd	4.6 ± 0.7 bc	4.2 ± 0.8 bc	2.8 ± 0.8 cd
*C. cautella*	CMW	4.2 ± 1.3 ab	1.9 ± 0.8 de	2.7 ± 1.2 de	3.8 ± 1.2 bc	1.3 ± 0.6 de	5.2 ± 1.0 a	1.2 ± 0.5 de
	FSF	1.1 ± 0.4 e	0.8 ± 0.2 e	3.1 ± 0.6 cd	2.5 ± 0.5 de	3.5 ± 0.6 bc	3.7 ± 0.8 bc	3.2 ± 0.8 cd
*E. kuehniella*	CMW	2.3 ± 0.9 a	1.4 ± 1.1 bc	0.8 ± 0.5 bc	1.0 ± 0.7 bc	0.5 ± 0.3 bc	1.3 ± 0.7 bc	0.4 ± 0.3 bc
	FSF	0.6 ± 0.2 bc	0.4 ± 0.2 c	1.2 ± 0.4 bc	1.2 ± 0.4 bc	1.5 ± 0.5 ab	1.4 ± 0.4 bc	0.8 ± 0.5 bc
*E. elutella*	CMW	1.1 ± 0.6 bc	1.3 ± 0.7 bc	1.5 ± 0.7 bc	1.9 ± 0.9 ab	1.3 ± 0.5 bc	1.8 ± 0.8 bc	0.7 ± 0.5 bc
	FSF	0.5 ± 0.2 bc	0.3 ± 0.2 c	1.1 ± 0.4 bc	1.6 ± 0.4 bc	2.1 ± 0.5 a	2.2 ± 0.6 a	1.4 ± 0.6 bc
*T. castaneum*	CMW	4.6 ± 2.2 ef	8.3 ± 7.2 cd	2.0 ± 0.9 ef	4.2 ± 1.7 ef	17.3 ± 5.1 ab	18.2 ± 5.7 a	10.3 ± 2.7 bc
	FSF	0.0 ± 0.0 f	0.0 ± 0.0 f	1.3 ± 0.4 ef	1.4 ± 0.4 ef	5.3 ± 1.1 de	5.0 ± 1.8 de	4.2 ± 1.1 ef
*T. confusum*	CMW	0.9 ± 0.6 b	1.1 ± 1.1 b	1.0 ± 0.4 b	0.8 ± 0.6 b	7.0 ± 2.0 a	7.5 ± 2.8 a	7.1 ± 2.2 a
	FSF	0.0 ± 0.0 b	0.0 ± 0.0 b	0.4 ± 0.2 b	0.6 ± 0.2 b	1.4 ± 0.4 b	1.3 ± 0.5 b	1.5 ± 0.6 b
*C. ferrugineus*	CMW	4.7 ± 2.0 bc	3.1 ± 1.4 bc	4.6 ± 2.4 bc	5.5 ± 2.1 bc	22.0 ± 9.9 a	9.2 ± 3.9 b	26.8 ± 8.9 a
	FSF	0.3 ± 0.2 c	0.3 ± 0.2 c	0.6 ± 0.2 c	1.4 ± 0.4 c	3.5 ± 2.9 bc	2.5 ± 0.7 bc	4.8 ± 0.8 bc
*A. advena*	CMW	1.8 ± 0.9 cd	1.8 ± 1.1 cd	0.8 ± 0.4 cd	2.1 ± 1.0 cd	4.5 ± 1.7 ab	2.4 ± 1.1 cd	6.8 ± 2.2 a
	FSF	0.3 ± 0.1 d	0.6 ± 0.2 d	0.3 ± 0.2 d	0.6 ± 0.2 d	2.3 ± 1.0 cd	2.1 ± 0.6 cd	2.6 ± 0.8 bc
*C. dimidiatus*	CMW	4.1 ± 2.0 a	3.3 ± 2.8 bc	0.8 ± 0.4 de	0.4 ± 0.3 de	1.3 ± 0.5 de	1.1 ± 0.5 de	3.5 ± 1.2 ab
	FSF	0.2 ± 0.2 e	0.2 ± 0.1 e	0.3 ± 0.1 e	0.2 ± 0.1 e	1.0 ± 0.3 de	2.0 ± 0.5 cd	3.9 ± 1.0 a
*T. stercorea*	CMW	0.0 ± 0.0 e	0.3 ± 0.3 de	0.7 ± 0.4 de	1.1 ± 0.4 de	3.1 ± 1.6 a	2.1 ± 0.9 bc	2.8 ± 1.0 ab
	FSF	0.0 ± 0.0 e	0.0 ± 0.0 e	0.2 ± 0.1 de	0.5 ± 0.2 de	1.4 ± 0.6 cd	1.5 ± 0.6 cd	1.8 ± 0.9 bc
*L. entomophila*	CMW	25.2 ± 9.7 ab	29.5 ± 5.2 ab	2.5 ± 1.3 de	8.0 ± 2.8 de	7.3 ± 3.6 de	3.1 ± 1.9 de	19.8 ± 5.6 bc
	FSF	1.3 ± 0.6 e	5.7 ± 1.4d e	10.9 ± 3.3 cd	7.1 ± 2.1 de	23.5 ± 4.5 b	8.6 ± 3.0 de	35.3 ± 10.1 a
*L. decolor*	CMW	20.8 ± 6.0 a	3.3 ± 2.0 cd	0.0 ± 0.0 d	0.0 ± 0.0 d	4.1 ± 2.8 cd	1.3 ± 0.9 d	13.8 ± 2.5 ab
	FSF	7.1 ± 1.5 cd	4.5 ± 1.2 cd	6.7 ± 2.9 cd	2.7 ± 0.9 d	17.3 ± 5.5 ab	5.5 ± 2.6 cd	8.5 ± 3.6 cd
*L. bostrychophila*	CMW	5.0 ± 2.1 bc	3.7 ± 1.9 bc	0.6 ± 0.4 bc	4.2 ± 1.9 bc	12.3 ± 7.0 ab	1.1 ± 1.1 bc	6.3 ± 3.8 bc
	FSF	0.8 ± 0.4 bc	0.0 ± 0.0 c	5.7 ± 2.6 bc	5.1 ± 2.1 bc	18.9 ± 4.9 a	7.9 ± 2.3 b	11.6 ± 3.7 ab
*L. pedicularia*	CMW	15.0 ± 7.3 a	0.8 ± 0.8 c	0.0 ± 0.0 c	0.0 ± 0.0 c	0.0 ± 0.0 c	0.0 ± 0.0 c	0.0 ± 0.0 c
	FSF	6.8 ± 1.5 b	2.6 ± 1.0 c	0.4 ± 0.2 c	1.2 ± 0.6 c	1.7 ± 0.8 c	0.0 ± 0.0 c	0.0 ± 0.0 c

## Data Availability

Data are contained within the article.
